# Assessment on latitudinal tree species richness using environmental factors in the southeastern United States

**DOI:** 10.7717/peerj.6781

**Published:** 2019-04-17

**Authors:** Youngsang Kwon, Taesoo Lee, Alison Lang, Dorian Burnette

**Affiliations:** 1 Department of Earth Sciences, University of Memphis, Memphis, TN, United States of America; 2 Department of Geography, Chonnam National University, Gwangju, South Korea

**Keywords:** Tree species richness, LASSO, Generalized linear model, Biodiversity, Peninsular effect, Florida

## Abstract

The southeastern region of the United States exhibits an unusual trend of decreasing tree species richness (TSR) from higher to lower latitudes over the Florida peninsula. This trend contradicts the widely marked latitudinal diversity gradient where species richness is highest in tropical zones and decreases towards extratropical regions. This study aims to assess the environmental factors that prompt this atypical inverse latitudinal gradient seen in TSR using the USDA Forest Service’s Forest Inventory and Analysis (FIA) database. Fifteen variables under four categories of forested area, groundwater, soil properties, and climate groups were examined to model TSR in the region. Generalized linear models (GLMs) with Poisson distributions first assessed individual variables to test explanatory power then the LASSO regularization method was utilized to extract two subsets of the most influential variables to predict TSR. Forest area and four climate variables (mean annual temperature, precipitation seasonality, mean temperature of coldest quarter, and mean precipitation of driest quarter) were the top five variables during the initial GLM assessment implying their potential individual influence in regulating TSR. Two subsets of LASSO models contained seven and three predictor variables, respectively. Frist subset includes seven predictors, presented in highest to low standardized coefficient, mean temperature of coldest quarter, forested area, precipitation seasonality, mean precipitation of driest quarter, water table depth, spodosol, and available water storage. The other subset further excluded four lowest influential variables from the first set, leaving the top three variables from the first subset. The first subset of the LASSO model predicted TSR with 63.4% explained deviance while the second subset reproduced 60.2% of deviance explained. With only three variables used, the second model outperformed the first model evaluated by the AIC value. We conclude that forest patch area, mean temperature of coldest quarter, and precipitation seasonality are the highly influential variables of TSR among environmental factors in the southeastern region of U.S., but evolutionary or historic cause should be further incorporated to fully understand tree species diversity pattern in this region.

## Introduction

Species richness, the number of different species within a defined area, depends on a variety of influences such as environmental factors, inter- and intraspecific interactions, evolutionary influences, and scale of observation (Dobzhansky, 1950; Hawkins et al., 2003; Pontarp & Wiens, 2017; Rohde, 1992). The US southeastern coastal plain was recently recognized as the 36th biodiversity hotspot by the Critical Ecosystem Partnership Fund (CEPF 2016). Although this region has more than 1,500 endemic vascular plants, its richest diversity is mostly herbaceous in the understory community of fire-dependent pine savannas and there’s a big knowledge gap in understanding the spatial patterns of arboreal stratum diversity (Noss et al., 2015). A latitudinal diversity gradient is a primary diversity pattern recognized in a wide spectrum of taxa in which the highest levels of species diversity are seen in the tropics while declining toward polar regions (Pianka, 1966; Rohde, 1992). Although this pattern is generally well understood at continent to global scale, the inverse trend is also present at the regional scale, but little is known about the evolutionary and environmental mechanisms that may cause this inverse trend.

Tree species richness (TSR) in the southeastern region of US forests exhibit an inverse latitudinal pattern (i.e., the TSR decreases from a mainland base towards the southern tip of the peninsula). Regarding evolutionary cause, Simpson (1964) postulated that the island-like geometry of peninsulas that are surrounded by ocean limit species immigration and increase extinction rates, causing a decrease in diversity from the base to the tip of the peninsula, also known as the peninsula effect. Although tree species has received little attention due to the lack of spatially explicit historic TSR records, a variety of mobile species with tracked movement attributed this pattern to peninsula effect; birds in Baja, California, and in Yucatan, Mexico (Wilson & MacArthur, 1967), and six groups of vertebrae on the Baja peninsula (Taylor & Regal, 1978). A study by Kwon, Larsen & Lee (2018) used surrounding waterbody area as an indirect measure for the peninsula effect, however, the influence of that variable was insignificant compared to other environmental variables. More direct investigation of peninsula effect of TSR was conducted by examining the latitudinal abundance patterns of 113 tree species assuming the steep abundance decline along the Florida peninsula is a ramification of past immigration–extinction dynamics (Kwon & Feng, 2019). However, most species (87% of 113 species) did not show an evidence for peninsula effect. Thus, although not conclusive, we assume that the peninsula effect (i.e., geometry caused immigration-extinction imbalance) has little impact on tree species in this study. An alternative explanation to the inverse latitudinal diversity pattern focuses on the varying environmental conditions of peninsulas (Jenkins & Rinne, 2008; Milne & Forman, 1986). In a study on woody plant diversity in Maine, Milne & Forman (1986) emphasized the importance of environmental heterogeneity in explaining inverse latitudinal diversity pattern. However, their study lumped data from multiple adjacent and small peninsulas in the region and focused on alpha diversity (i.e., plot-level measures of diversity) rather than landscape-level gamma diversity adopted in this study. In a study on breeding bird diversity on the Baja peninsula, Wiggins (1999) determined that local habitat heterogeneity had a greater influence on bird diversity than immigration rates on Baja Peninsula. Means & Simberloff (1987) also examined richness of amphibians and reptiles in Florida and found that the highest levels of richness were seen along the mid region of the peninsula attributed by habitat preference on environmental factors rather than evolutionary cause. Other hypotheses predict history may influence current richness trends as species may still be colonizing the peninsula or the current richness patterns may reflect historic sea-levels (Seib, 1980).

The TSR studies seeking a causal mechanism of environmental factors are a challenging task because tree species exhibit a delayed reaction to the environment in their demographic processes requiring long term monitoring with an expensive sampling campaign covering a large geographic extent.

The purpose of this study is to comprehensively evaluate climate variables, forest size, ground water, and soil properties that have all demonstrated potential in determining TSR in other regions. The ground water and soil properties were especially included due to the large spread of swamps and woody wetlands in the region. These environmental conditions potentially vary across the peninsula landform and may limit the tree species that can tolerate those conditions, resulting in a decrease in TSR across the peninsula. We provide a brief review of the selected group of environmental factors in the following sub-section to support our variable selection.

### Literature reviews on selected environmental factors

#### Climate

Temperature and precipitation are the two most influential abiotic factors on the distribution of plant species (Prentice et al., 1992). Francis & Currie (2003) compared global patterns of species richness to climate and revealed mean annual temperature, annual water deficit, and annual potential evapotranspiration to be strongly correlated to taxonomic group richness. However, the geometry of Florida may result in different climate patterns than those seen in the mainland due to the surrounding water bodies. Warmer waters surrounding the coast of the peninsulas can increase the amount of precipitation over a region, as warmer waters provide the atmosphere with larger amounts of moisture which eventually condenses in the atmosphere and becomes precipitation. These differences in climate of the peninsula may limit the tree species that can inhabit the region. Severe climatic events such as droughts can also have a negative impact on species richness (Bigler et al., 2007; Dale et al., 2001). Martínez-Vilalta & Piñol (2002) examined drought induced mortality of pine populations in the Iberian Peninsula and found varying influence of drought depending on species’ water-use efficiency. A large portion of south Florida is a fire-maintained ecosystem (e.g., pine savannas). Although the influence of fire history (both wild and prescribed fire) to TSR is lacking, a drought index may also imply the frequency of wild fire.

#### Forested area

The species richness and area relationship (species–area curve) is a well-known phenomenon (Chisholm et al., 2013; Williamson, Gaston & Lonsdale, 2001). A larger area can support more diverse habitats and larger quantities of resources while maintaining higher immigration rates and lower extinction rates due to the greater number of individuals able to thrive in the area. TSR is related to the size of the study area if TSR is derived from irregularly sized study areas (Latham & Ricklefs, 1993; Belote et al., 2011). However, even when TSR is derived from the regularly sized grids (i.e., standardized by area), forest patch area may still vary due to presence of other covers, thus a species - area relation might remain important factor in gridded TSR data (Kwon, Larsen & Lee, 2018).

#### Ground water

The southeastern regions of the United States contain large tracts of marshes, swamps, and woody wetlands that result in extremely shallow water tables which may potentially limit TSR. While shallow water tables may limit TSR, so can the opposite condition. Deep water tables can potentially hinder tree growth and survival if their roots don’t have access to adequate water in the soil. The water table can have localized cones of depression due to tree clusters and pumping wells (Yang, McMillan & Zammit, 2017). Zinko et al. (2005) found a positive correlation between TSR and the availability of shallow groundwater in predominantly dry area in Sweden. They found areas of shallow groundwater had higher TSR with rare plant species suggesting that groundwater may be a strong determinant of richness in regions where groundwater availability is a limiting factor. However, there is limited empirical study on TSR in predominantly shallow water table areas.

#### Soil properties

Plants depend on soils for support, nutrition, and water storage. In the study of Fan & Waring (2009), at the broad ecoregional scale, water storage in the soil was the most dominant variable in predicting TSR in two of the ecoregions, the Southeastern Plain and Middle Atlantic Coastal Plain. Also, Prentice et al. (1992) incorporated available water storage into a global vegetation prediction model for predicting biomes and their corresponding vegetation and found the water storage variable as an essential predictor in their model along with minimum temperature. The large portion of Southern Coastal Plain is covered in sandy Spodosol (e.g., Myakka as state soil for Florida), but its drainage capacity can vary widely. The study area includes nine of 12 major global soil orders and with soil pH ranging from 3 to 8.5 with a high pH due to the influence of the limestone bedrock in south Florida (Noss et al., 2015).

## Materials & Methods

### Study area

The study area is comprised of South Carolina, Georgia, Alabama, and Florida, which encompasses a variety of forest types, land covers, and landforms. This permits the assessment of environmental factors that influence the range of TSR from very low richness in southern Florida to very high in northern Georgia and Alabama. Large tracts of woody wetlands with shallow water tables are primarily seen along the coast and following waterbodies farther inland. The dominate forest types include oak-hickory, loblolly-shortleaf pine, and longleaf-slash pine. The oak-hickory group is seen in the northern region of the study. The loblolly-shortleaf pines cover regions in the Outer Coastal Plain and the Southeastern Mixed Forest provinces ranging from on the coast to farther inland. The longleaf-slash pine is found mainly in the Outer Coastal Plain in southern Alabama and Georgia, and northern Florida. The presence of gum-cypress and pine forests interspersed suggests the presence of xerophytic and hydrophytic forms of habitats, either excessively dry or wet conditions (Bailey et al., 1994).

### Forest Inventory and Analysis (FIA)

The FIA program is a nation-wide strategic forest survey that originally served as the foundation of a continental-scale policy study required by Resources Planning Act (RPA). Forest Inventory and Analysis Database (FIADB version 6.0; https://apps.fs.usda.gov/fia/datamart/datamart_access.html) used in this study is the most recent complete five-year (2011–2015) cycle of inventory. The FIA program has implemented a common plot design comprised of four 24-foot radius subplots where all trees with a diameter greater than five inches are identified and measured. Each subplot houses a 6.8-foot radius microplot where trees with a diameter of less than five inches are measured. The study area includes over 14,000 FIA plots across 545,500 km^2^. The FIA dataset of four states (Alabama, Florida, Georgia and South Carolina) was downloaded as Microsoft Access Databases from the Forest Service’s FIA DataMart and four tables (table name in the FIADB: Plot Snapshot, Population Evaluation Group, Condition, and Tree) were used to estimate TSR. The 2015 population evaluation groups (Inventory year from 2011 to 2015) were selected for each state to ensure the timing of sampling events to be consistent across all four states. About 15% of plots in the study area showed forest management practice activity such as artificial regeneration, cutting and clearing thus we excluded those plots using the FIADB code of TRTCD during the examined five-year inventory cycle. As a result, a total of 511,903 tally trees from 11,680 inventory plots were used across the study area. A plot-level TSR estimation (see ‘Tree Species Richness’ below) and 15 environmental variables (see ‘Environmental variables’ below) were aggregated into 20 km by 20 km grids (total 1,312 grids). This grid size is chosen to match area of FIA sampling framework (Kwon & Larsen, 2013; McRoberts et al., 2005). Issues with perturbed (fuzzed and swapped) FIA plot locations representing privately-owned land were negligible in this study, as they were aggregated over a much larger area (20 km by 20 km) of grids (Gibson et al., 2014; Prisley et al., 2009). All environmental variables were aggregated at the grid unit as a mean value, except the categorical variables of soil hydrologic group and Spodosol where the majority values are used.

### Tree Species Richness (TSR)

TSR, counts of unique tree species, was estimated by a bootstrapping method following Kwon, Larsen & Lee (2018) to ensure our sample-based TSR estimates were not biased by the number of plots at the grid unit because the relationship between the number of plots and the TSR in a grid was linear (Pearson’s *r* of 0.58, *p* < 0.01). The number of plots per grid ranged from 1 to 23 plots with an average of 13.5 plots per grid. We first selected only grids that contained more than three plots and then we applied bootstrapping methods of 1,000 iterations to calculate mean values of species counts (TSR) after randomly selecting three plots for each grid.

### Environmental variables

Fifteen environmental variables (predictor variables) were employed and grouped into four categories that may influence TSR: forested area, groundwater, soil properties, and climate (Table 1).

**Table 1:** Grouping and description of 15 predictor variables used in the model. Spodosol, which was used as a categorical dummy variable and not included in the initial GLM analysis.

Group	Predictor variable	Rank of influence by individual GLM	Standardized coefficient	Deviance explained (%)	Data source
Forested Area	Forested Area (FA)	1	0.346	46.23	MODIS
Groundwater	Water Table Depth (WTD)	6	0.198	21.72	Fan, Li & Miguez-Macho (2013)
Soil Property	Available Water Storage (AWS)	7	0.136	8.35	gSSURGO
	Soil Hydrologic Group (SHG)	8	0.120	7.37	
	Spodosol	–	–	–	
Climate	PET	12	−0.173	10.41	MODIS
	AET	9	−0.100	3.68	
	Mean Annual Temperature (MAT)	2	−0.302	41.91	WorldClim
	Mean Temperature of Coldest Quarter (MTCQ)	4	−0.331	44.60	
	Mean Annual Precipitation (MAP)	10	0.168	8.35	
	Precipitation Seasonality (PSN)	3	−0.360	44.71	
	Mean Precipitation of Driest Quarter (MPDQ)	5	0.233	26.73	
	Temperature Seasonality (TSN)	11	0.146	7.98	
	SPEI 1994–1999 (SPEI I)	14	0.001	0.00	WestWide Drought Tracker
	SPEI 1999–2004 (SPEI II)	13	0.015	0.11	

Forested area (FA) variable was obtained from the remotely sensed MODIS satellite product (MOD12, collection 5) and downloaded from the USGS Land Processes Distributed Active Archive Center Data Pool (https://lpdaac.usgs.gov/data/). We used IGBP land cover classification scheme (Friedl et al., 2010) to calculate total areas of forest related land covers (evergreen needleleaf, evergreen broadleaf, deciduous needleleaf, deciduous broadleaf, and mixed forest) within each grid. The water table depth (WTD) variable under the ground water group was downloaded as a NetCDF file with a raster resolution of 30 arc-seconds (approximately 1 km resolution) from USGS (https://waterdata.usgs.gov/nwis). The WTD is a simulated dataset based on water level measurements, ground water flow and long-term table solution of the balance climate fluxes by Fan, Li & Miguez-Macho (2013). The data set was validated by Fan, Li & Miguez-Macho (2013) with over 500,000 field observations of water table depth performed by the USGS between 1927 and 2009. The mean of the residuals was 0.443 m (Fan, Li & Miguez-Macho, 2013). The soil property group includes three variables, Available Water Storage (AWS), Soil Hydrologic Group (SHG) and Spodosol layer, from the gSSURGO (Gridded Soil Survey Geographic) database downloaded from the USDA Natural Resources Conservation Service (NRCS) (https://datagateway.nrcs.usda.gov). The AWS is the volume of water that the soil can store, measured in cm to the depth of 150 centimeters, that is available to plants. We used the SHG classification that categorizes soils into four main classes (A, B, C, and D) based on their infiltration and runoff characteristics, which largely correspond to soil texture and composition from high infiltration rate/low runoff potential (Group A) to low infiltration rate/high runoff potential (Group D) in Table 2. The SHG variable is used to represent soil types in addition to water infiltration rate, as it is strongly correlated to soil types. Lastly, to account for the potential influence of the presence of a hardpan in the Spodosol—a soil order abundant in Florida—which may hinder root growth, we used Spodosol soil order maps as a categorical dummy variable.

**Table 2:** The basic characteristics of the soil hydrological groups (SHG), which are based on infiltration rate, water transmission, and runoff potential.

SHG	Soil type	Infiltration rate and water transmission	Runoff potential
A	Sand, gravel, loamy sand, sandy loam	High	Low
B	Silt loam, loam	Moderate	Moderate
C	Sandy clay loam	Moderate	Moderate
D	Clay loam, silty clay loam, sandy clay, silty clay, clay	Low	High

The climate group includes ten variables (Table 1). We used 30 arc-second (ca. 1 km at the equator) resolution of monthly temperature and precipitation data for the time period 1950 to 2000 from the WorldClim data set (http://www.worldclim.org) to calculate the following six variables: Mean Annual Temperature (MAT, °C), Mean Temperature of Coldest Quarter (MTCQ, °C), Mean Annual Precipitation (MAP, mm), Mean Precipitation of Driest Quarter (MPDQ, mm), Precipitation Seasonality (PSN, mm), calculated as the standard deviation of the monthly precipitation totals divided by the mean monthly precipitation, and Temperature Seasonality (TSN, °C) as standard deviation of the mean monthly temperature data. Two other climate variables, potential evapotranspiration (PET, mm) and actual evapotranspiration (AET, mm), were downloaded from the MODIS Global Evapotranspiration Project (http://www.ntsg.umt.edu) for the period 2000–2010 with a 1 km by 1 km spatial resolution. PET is a surrogate for the net atmospheric energy balance independent of water availability and AET is the amount of water that was actually removed from a surface. Lastly, the drought index of Standardized Precipitation Evapotranspiration Index (SPEI) was downloaded from the West Wide Drought Tracker. We downloaded for two five-year periods of SPEI; 1994–1999 namely as SPEI I and 1999–2004 as SPEI II because trees exhibit a lag effect to changes in environmental conditions. If a drought did affect tree distribution by inhibiting successful reproduction or the growth of saplings into mature trees (mortality), then the affects would be seen seasons to years later (Bigler et al., 2007).

### LASSO regularization of generalized linear model

The generalized linear model (GLM) has been utilized in many species richness studies (Buse & Griebeler, 2012; Guisan & Zimmermann, 2000) and its ecological applications fully reviewed by McCullagh & Nelder (1989). The Least Absolute Shrinkage and Selection Operator (LASSO; Tibshirani, 1996) model was enacted under the generalized linear model (GLM) specification for its ability to select variables that are the most important from highly correlated variables through its shrinkage and selection method for linear regression. LASSO is a continuous selection algorithm that selects a subset of predictors by shrinking the coefficient of unimportant ones to zero and eliminating them from the algorithm; making LASSO ideal for dealing with multicollinearity in data (Tibshirani, 1996). The environmental variables used as predictor variables in this study are highly correlated with one another. For example, precipitation and temperature trends affect the regional drought index and water table depth. All continuous predictor variables were standardized (mean = 0, SD = 1) so that the regression coefficients could be compared as measures of relative importance (Schielzeth, 2010). The Spodosol variable, a categorical variable, was added as a dummy variable in the model. We first ran GLMs with a log link function for each of our single predictor variables (except the categorical Spodosol variable) to evaluate their individual explanatory power. Then we performed LASSO GLM with 10-fold cross-validation to determine the optimal regularization penalty parameter, *λ* value, to use in the prediction models. The *λ* values were chosen to determine which predictor variables to incorporate in creating the prediction models. We used two values of *λ* for selection; one is the *λ* minimum (LASSO *λ* min) that provides the minimum cross-validated error and the other is the *λ* 1 standard error (LASSO *λ* 1se) that provides the regularized model where the error is within one standard error. Both *λ* values were used to produce prediction models of TSR as the selected predictors were different for each *λ* value used. The two LASSO GLMs were compared by variance inflation factors (VIFs), model performance as indicated by AIC and percent of total deviance explained. VIFs indicate if multicollinearity exists in a regression analysis by examining how much the variance of estimated regression coefficients are inflated as compared to when predictor variables are not linearly related. Total deviance explained was calculated by the difference between the deviance for the given model and the saturated model as:

(1) \begin{eqnarray*}& & 2\sum _{i=1}^{n}{y}_{i} \left( \log \nolimits \left( \frac{{y}_{i}}{{u}_{i}} \right) - \left( {y}_{i}-{u}_{i} \right) \right) .\end{eqnarray*}

Then, the percent deviance explained was calculated as (100-null deviance/residual deviance) * 100.

The LASSO procedure was conducted under the R version 2.12.1 (R Core Team, 2014) environment mainly using the *glmnet* package (Friedman, Hastie & Tibshirani, 2010).

## Results

### Explanatory assessment of TSR and predictor variables

**Figure 1:**
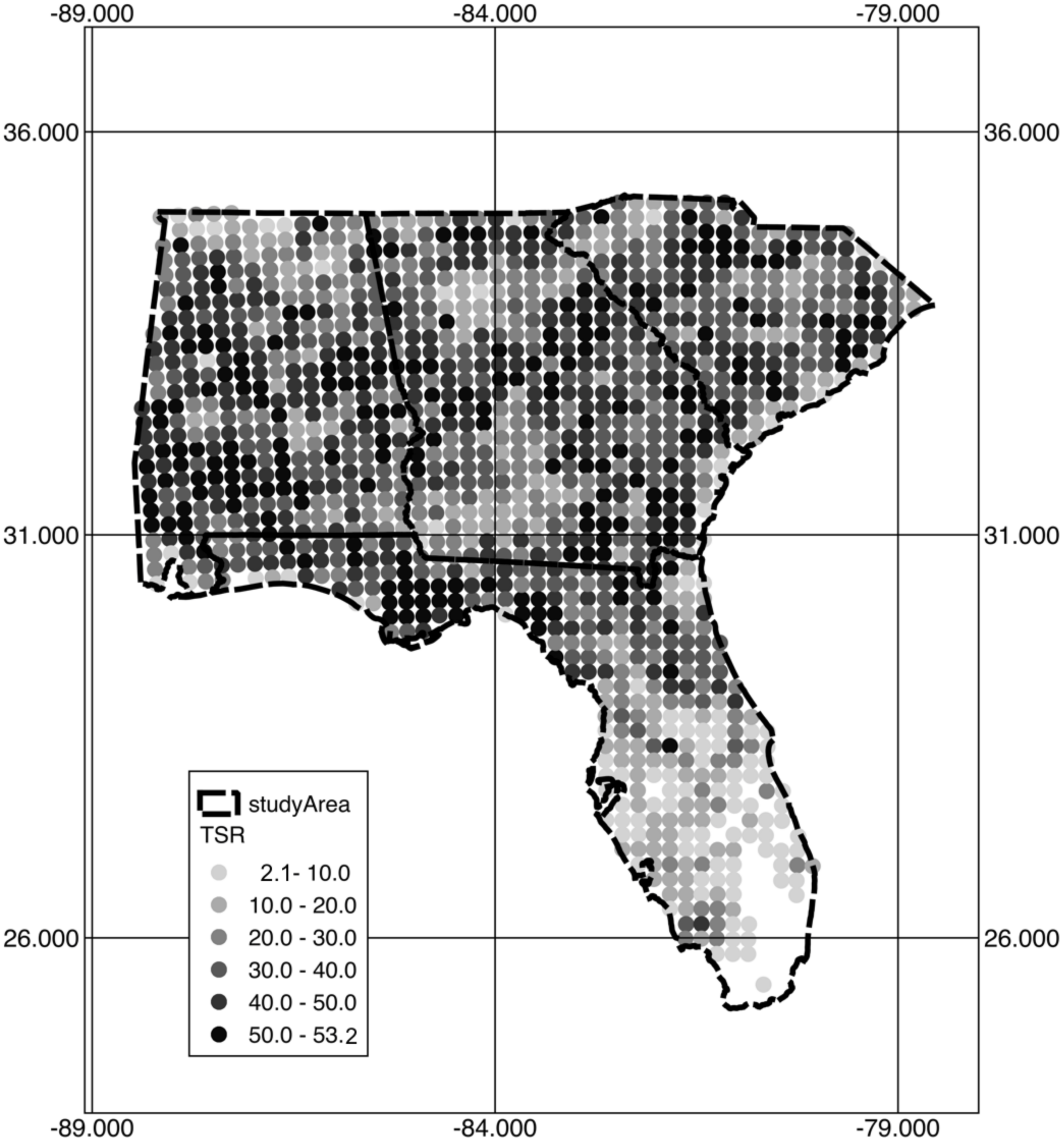
Observed TSR via the bootstrapping method.

The observed TSR values, response variable, via a bootstrapping method exhibited a mean TSR value of 20.1 (median TSR of 21) in the study area. The TSR values were highest (53.2) in the northern areas of Georgia and South Carolina and throughout Alabama and lowest (2.1) at the tip of the Florida peninsula and at the boundary between Georgia and Florida (Fig. 1).

**Figure 2:**
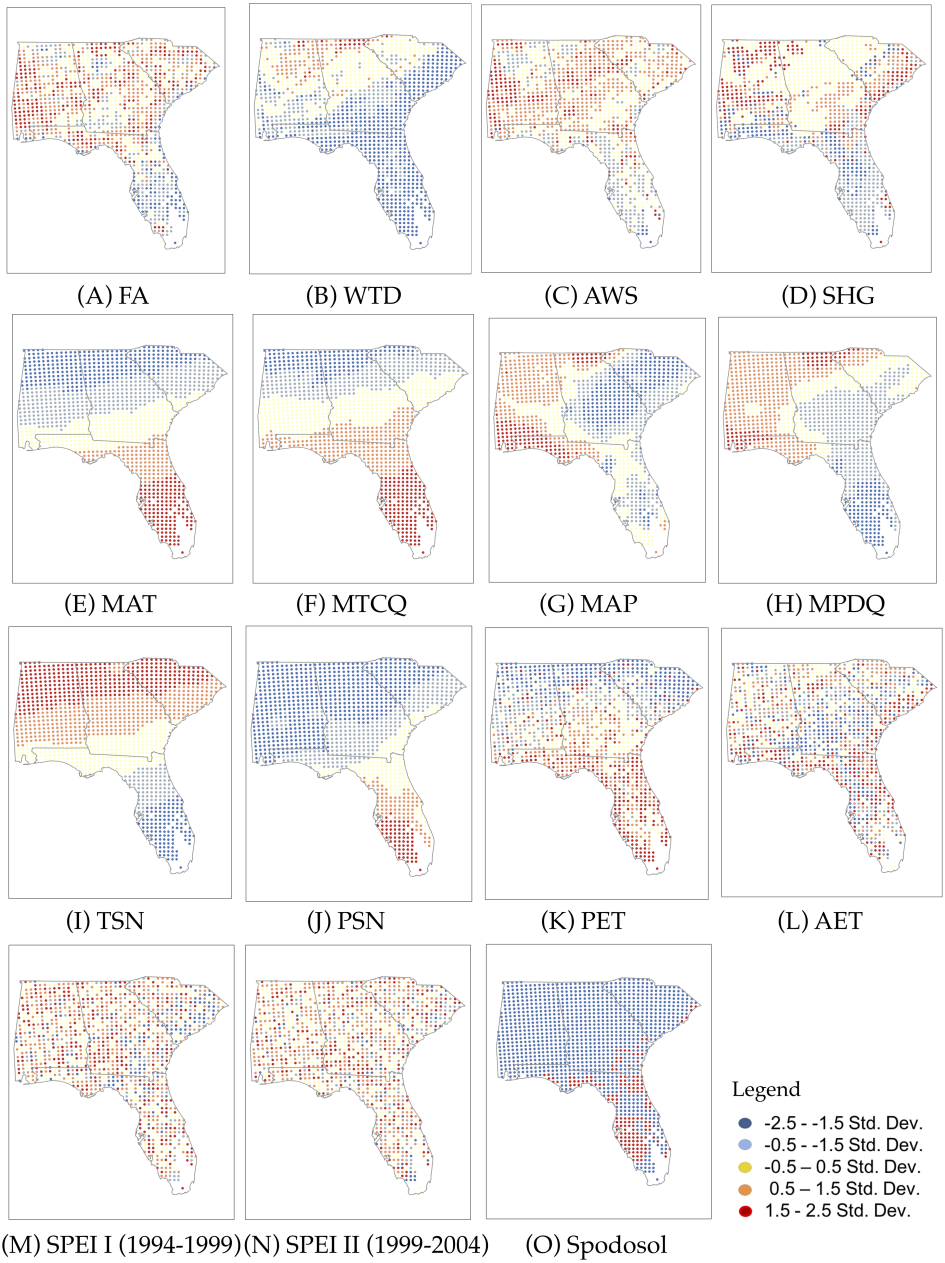
Geographic patterns of 15 predictor variables (A–O). Five classes of standard deviations (Std. Dev.) are commonly used to map the spatial variation of each variable.

Figure 2 depicts 15 predictor variables (14 variables classified by their standard deviations and a categorical variable of Spodosol). The description of geographic patterns of predictor variables are provided in the order presented in Fig. 2. The lower latitudes of the Florida peninsula have noticeably sparser FA than the rest of the study area. Regions of high FA are seen in coastal areas, like the Florida panhandle, and farther inland. The WTD showed a shallow water table for the majority of Florida and the coastal plain regions of Georgia and South Carolina. The southern and coastal regions showed WTD between 0.1 m and 10.2 m below the surface. Deeper water tables reaching up to 50 m deep were observed at the northern state lines of Alabama, Georgia, and South Carolina. The average AWS ranged from 0 cm to 35 cm across the study area. The northern areas of Alabama and Georgia showed areas of low AWS, which may be due to soil type. Florida had a low water storage along the coastal regions with a mix of high-water storage in interior Florida. The study area contained all seven groups of SHG. Converted to integer values from 1 to 4, Florida is comprised of low SHG values reflecting mostly groups A, which correspond to areas of shallow water table depth. Coastal regions of South Carolina exhibited high SHG values, representing group D. The MAT and MTCQ ranged from 10 °C to 24 °C and 8 °C to 19 °C, respectively. The southern tip of Florida exhibited the highest MAT, while the lowest values were observed over northern Georgia where it is mountainous. The MTCQ exhibited a similar geographic pattern to MAT but the latitudinal pattern was weaker than MAT. The MAP showed an overall high value in the Florida panhandle and Alabama, while low values were observed in central Florida, Georgia and South Carolina. While MPDQ showed a similar pattern to MAP, low values were observed throughout Florida with the lowest values found on the southern tip of Florida. TSN increased as latitude increased similar to MAT, while PSN showed an inverse pattern where the highest values were found on the southern tip of Florida, especially the west side which may be due to its proximity to the warm waters of the Gulf of Mexico. PET showed, in general, a decreasing pattern with latitude following the MAT gradient, while AET exhibited a more scattered pattern with pockets of high values near coastal area and interior Florida. Both SPEI I (1994–1999) and SPEI II (1999–2004) showed local scale mixed patterns compared to the regional scale patterns detected with the other climate variables. Spodosol was found mostly in coastal Florida and concentrated over the southwestern portion of Florida.

### Model prediction

Individual predictor variables assessed by the initial GLM showed five predictor variables that explained >25% of the deviance in TSR (presented in descending order): FA, MAT, PSN, MTCQ, and MPDQ (Table 1) with MAT, PSN, and MTCQ being negatively related to TSR. The absolute value of the standardized coefficient followed the same order as did the deviance explained. The LASSO regularization method produced two sets of variables chosen by *λ* min (0.00143) and *λ* 1se (0.00315) via 10-fold cross-validation. Out of a total of 15 variables, the first variable set (LASSO *λ* min), selected by the *λ* min, included six variables - presented in descending order of absolute standardized penalized coefficient - MTCQ, FA, PSN, MPDQ, WTD, Spodosol and AWS, while the other variable set (LASSO *λ* 1se), selected by the *λ* 1se, contained three variables—FA, MTCQ and PSN in descending order of absolute standardized penalized coefficient (Table 3). VIF values for all variables in the two models were less than five, indicating negligible multicollinearity among predictor variables selected in both models.

**Figure 3:**
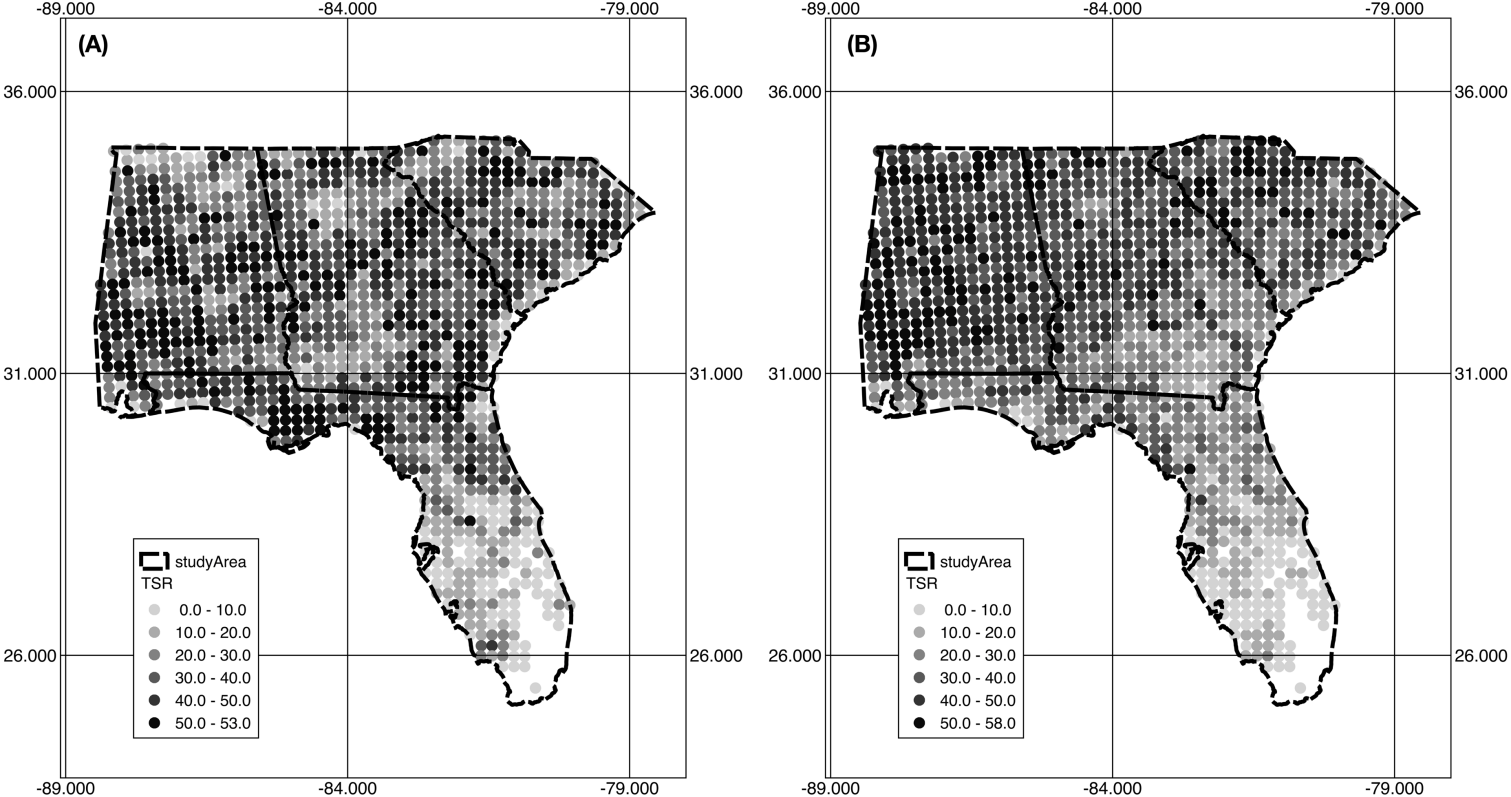
TSR prediction by the LASSO *λ* min model (A) and the LASSO *λ* 1se model (B).

**Table 3:** Selected variables by two LASSO GLM models. Variables are ordered from highest to lowest absolute standardized coefficient.

LASSO *λ* min model			LASSO *λ* 1se model		
Selected variables	Standardized penalized coefficient	VIF	Selected variables	Standardized penalized coefficient	VIF
MTCQ	−0.0030743	3.45	FA	0.0044581	1.46
FA	0.0025398	1.22	MTCQ	−0.0036412	1.25
PSN	−0.0002472	1.26	PSN	−0.0002364	1.67
MPDQ	0.0012111	2.27	–	–	–
WTD	0.0001551	4.62	–	–	–
Spodosol	−0.0001271	3.67	–	–	–
AWS	0.0001199	2.86	–	–	–
AIC	7,378		7,195		
Null deviance	3,559		3,559		
Residual deviance (% explained)	2,256 (63.4%)		2,043 (60.2%)		

The LASSO *λ* min model exhibited a slightly higher % deviance explained (63.4%) than the LASSO *λ* 1se model (60.2%), however, the AIC value, which considered the number of variables selected was lower for the LASSO *λ* 1se model. Thus, the LASSO *λ* 1se model outperformed LASSO *λ* min. The amount of % deviance explained by the two variable subsets differed only slightly, suggesting four variables (MPDQ, WTD, Spodosol and AWS) not included in LASSO *λ* 1se model added only marginal explanatory power.

Figure 3 shows the predicted TSR values modeled by LASSO *λ* min and LASSO *λ* 1se. The two sets of predicted TSR appear to have a very similar geographic pattern: both models produced a smooth change in TSR that predicted TSR decreases closer to coastal areas and with lower latitude on the peninsula. The predicted TSR maps, however, failed to capture the scattered regions of mid to high levels of observed TSR seen along the Florida panhandle but overall produced a smooth inverse latitudinal diversity pattern that mirrors the observed TSR gradient. The two models predicted TSR comparable to observed TSR with slightly higher TSR for LASSO *λ* 1se (Table 4).

**Table 4:** Predicted TSR by LASSO *λ* min and LASSO *λ* 1se models compared to observed TSR.

	LASSO *λ* min	LASSO *λ* 1se	Observed TSR
Min	1.1	1.5	2.1
Mean	22.2	23.3	20.1
Median	20.5	21.4	22
Maximum	52.9	57.8	53.2

## Discussion

### Forested area

The FA variable was the strongest individual variable (46.23% deviance explained) in the initial GLM and the second and first strongest variable in LASSO *λ* min and LASSO *λ* 1se model, respectively. This result supports the species–area hypothesis, where TSR is largely influenced by the size of forest patches. Southern Florida has limited forested area according to the FIA Phase 1 standards of forest classification (at least 1 acre in size with at least 10% stocked with tree species). Thus, only a few plots were installed on the Florida Keys and the southern tip of the peninsula. Gillespie (2005) aimed to fill this data gap by predicting stand-level TSR in the tropical dry forests using landscape variables such as area, nearest neighbor distance, and boundary complexity and forest patch area still proved to be a highly influential variable in southern Florida just as seen in our LASSO variable selection of the larger study area. Perhaps an approach to incorporate local studies of TSR with the FIA data needs to be formulated to better understand diminishing habitats like the tropical dry forest found on the southern tip of the Florida peninsula.

### Groundwater

The WTD variable was the sixth in the initial GLM (21.72% deviance explained) and fifth most influential variable included in only LASSO *λ* min model showing potential in predicting TSR in the region. Large portion of coastal areas are composed of marshes, wetlands or swamps and were initially hypothesized to limit TSR. Generally, low TSR values were seen in areas containing a shallow water table, which advocates for the potential influence of the shallow water table on richness. Previous research on plant richness trends in arid environments found groundwater to be a limiting factor and a highly influential variable (Allen-Diaz, 1991; Horton, Kolb & Hart, 2001). Groundwater is expected to have a stronger influence on richness in arid environments where water is limited but this study suggests groundwater has a marginal influence on TSR in temperate, humid regions. This study did not assess the influence of seasonal changes in the water table on TSR due to the lack of available data but researchers have found the seasonal variation in WTD to influence TSR and tree mortality (Allen-Diaz, 1991; Braun et al., 2004). In addition, the simulated WTD may not accurately depict areas of localized depression due to agricultural pumping although localized depressions would not likely have affected this study’s results due to the methodology of averaging the predictor variables by grid cell.

### Soil properties

The soil properties demonstrated a small degree of influence on TSR in the study area. Both AWS and SHG demonstrated a moderate relationship to TSR when assessed by initial GLMs (7th and 8th rank, respectively). The Spodosol and AWS variable were included as sixth and seventh in the LASSO *λ* min model, respectively, proved to be more influential than the SHG variable that was not included in both models. Research assessing the influence of AWS and SHG has been limited but soil properties may be more influential in other regions with different environmental constraints and tree species. For example, Beedlow et al. (2013) found the AWS had a strong effect on the growth of Douglas-firs in Oregon. Unlike the expectation, WTD and two soil predictor variables (AWS and SHG) were loosely related: Pearson’s correlations between WTD-AWS, WTD-SHG and AWS-SHG were 0.11, 0.16 and 0.20, respectively. The three variables represent water availability to trees either as the water table (WTD), water between soil pore spaces (AWS), and in the water’s ability to infiltrate the soil (SHG). The limitation with the AWS variable was that it only exhibited minor, gradual changes between grid cells across the study area. Spodosol in Florida, if containing a hardpan layer—usually 80–100 cm below the surface- may hinder the growth of tree roots beyond hardpans. However, the Spodosol map used as categorical variable had little variation within it and showed only a moderate influence in the model. Also, many pedological observations suggested that the Bh horizon in spodosol do not act as hardpans, not always restrictive of root penetration, and typically do not have low permeability (Jenny, 1980).

### Climate

Four climate variables were the second to fifth strongest variables (MAT, PSN, MTCQ, and MPDQ in descending order of standardized coefficient) assessed by the initial GLMs. Among them, MTCQ and PSN were the first and third, and second and third strongest variables selected by LASSO *λ* min and LASSO *λ* 1se model, respectively. Climate variables, such as temperature and precipitation, have been the focal point of several latitudinal diversity gradient mechanisms (Currie, 1991). Although MAT exhibited the second most explanatory power by the initial GLM, when assessed by LASSO, it was not selected due to the strong correlation with MTCQ (Pearson’s r of 0.91, *p* < 0.01). Although LASSO handled multicollinearity well, it was indifferent to variables with very strong correlations and chose one of the highly correlated variables and ignored the others (Friedman, Hastie & Tibshirani, 2010). Because Florida is the most lightning-prone states in the US (Grimm et al., 2006), we use a drought index as a proxy for fire. The little influence of drought index may due to a lagged effect of tree mortality to changing environment (Bigler et al., 2007). Although the drought indices (SPEIs) showed little influence on TSR, the MPDQ variable, fifth strongest variable by initial GLM, still demonstrated that precipitation in the dry season is an important factor in limiting TSR. Crumpacker, Box & Hardin (2001) also predicted extensive disruption to major woody ecosystems in Florida over a 100-year period of warming scenarios, where the climate models predicted an increase in annual temperature of 1° to 2 °C and a change in annual mean precipitation from −20% to +10%. Studies using fossil pollen records in this region showed remarkable climatic stability over long periods (Grimm et al., 2006), and some fire-dependent pine savannas have analogs extending back to the Eocene, maintained mainly by stable natural processes (Graham, 1999). However, a large portion of forested areas are converted from savannas and woodlands prior to European colonization through active fire suppression. Although we could not implement successional stage as a variable in our model, those areas may be still experiencing the early successional stages. Thus, they are expected to have lower diversity than late successional stage forests.

## Conclusions

The inverse latitudinal diversity gradient of TSR seen from northern Georgia and Alabama to the southern tip of the Florida peninsula is strongly influenced by a combination of forested area, minimum temperature of the coldest quarter, and precipitation seasonality. Marginal influences by groundwater (water table depth) and soil properties (available water storage) were also detected. Among those environmental variables shown influential to TSR, forested area is the only variable that can be quickly altered due to anthropogenic pressures. The other climatic, groundwater and soil property variables are also affected by anthropogenic pressures, but changes are typically very gradual. Although, it is not possible to suggest how much forested area is required to impact TSR without considering variables interactions, it is noteworthy that 46% of deviance was explained in the GLM by using only the forested area variable. Thus, by identifying forest patch area as a highly influential variable of TSR in the study area, forest and land managers should try to preserve and increase forest patch sizes to help combat the anticipated effects of climate change on TSR.

##  Supplemental Information

10.7717/peerj.6781/supp-1 Dataset S1 Environmental Variables

## Additional Information and Declarations

### Data Availability

The following information was supplied regarding data availability: The raw data is available as Supplemental File.
